# Supplementary material for: Encoding and Multiplexing Information Signals in Magnetic Multilayers with Fractional Skyrmion Tubes
Source: ACS Appl Mater Interfaces. 2023 Jul 10;15(28):34145–58. doi: 10.1021/acsami.3c01775 (PMC10360071; doi:10.1021/acsami.3c01775)
Supplement: Supplementary file 2 — am3c01775_si_002.pdf [file am3c01775_si_002.pdf]

Supporting Information

Encoding and multiplexing information signals in magnetic multilayers with fractional skyrmion tubes

Runze Chen[1], Yu Li[1], Will Griggs[1], Yuzhe Zang[1], Vasilis F. Pavlidis[2], and Christoforos Moutafis[1*]

[1] *Nano Engineering and Spintronic Technologies (NEST) research group, Department of Computer Science, The University of Manchester, Manchester M13 9PL, United Kingdom*

[2] *Advanced Processor Technologies (APT) research group, Department of Computer Science, The University of Manchester, Manchester M13 9PL, United Kingdom*

[*]corresponding author: christoforos.moutafis@manchester.ac.uk

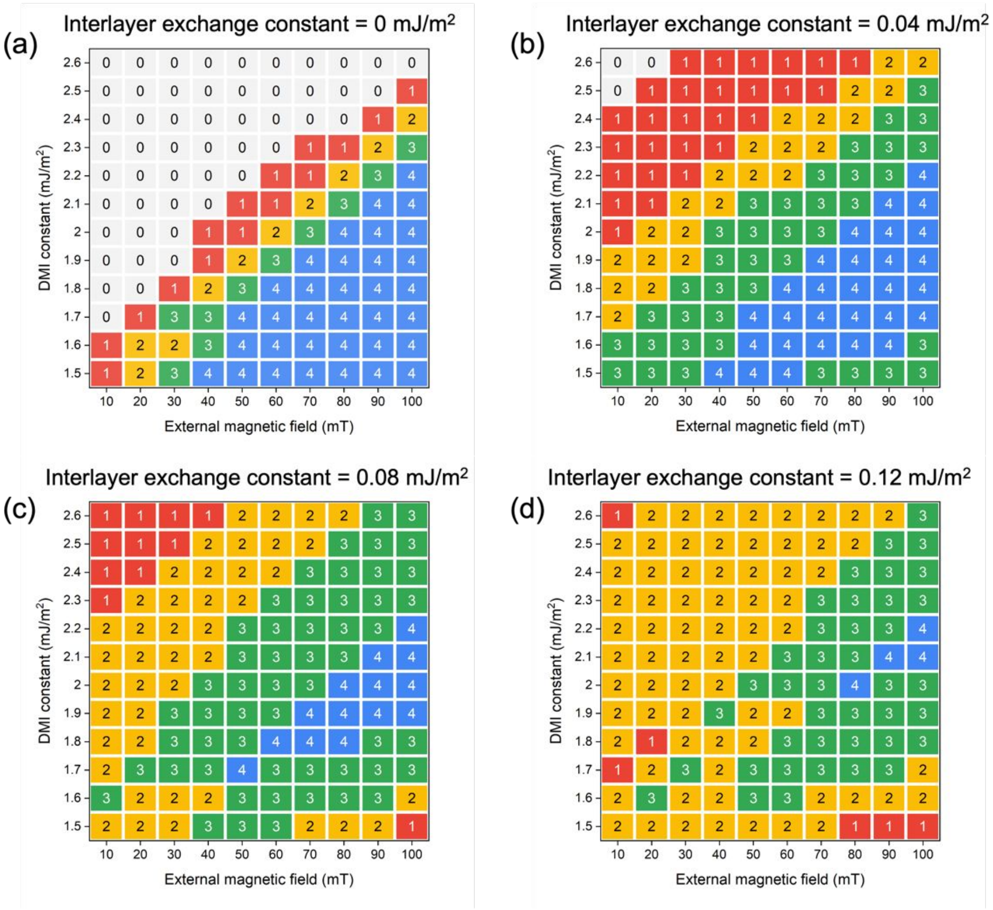

Figure S1. Distinct FST states phase diagram in 4 MMLs system with different interlayer exchange coupling. Phase diagram of the number of distinct stable FST states with various external out-of-plane magnetic fields and DMI constant in a four magnetic layers system with the interlayer coupling of (a) 0 mJ m^{-2}, (b) 0.04 mJ m^{-2}, (c) 0.08 mJ m^{-2}, and (d) 0.12 mJ m^{-2}, respectively. The colour code is the same as in Fig. 1(b) in the main text, representing the number of supported distinct states.

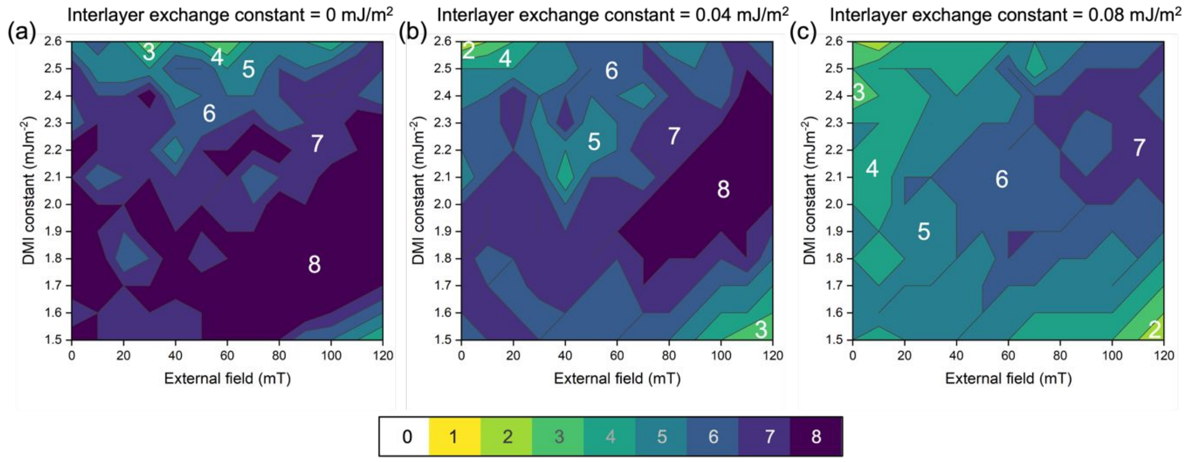

Figure S2. Distinct FST states phase diagram in 8 MMLs system with different interlayer exchange coupling. Phase diagram of the number of distinct stable FST states as a function of out-of-plane magnetic field and DMI constant in an 8MML system with the interlayer coupling of (a) 0 mJ m^{-2}, (b) 0.04 mJ m^{-2}, and (c) 0.08 mJ m^{-2}, respectively. The colour code represents the number of supported distinct states.

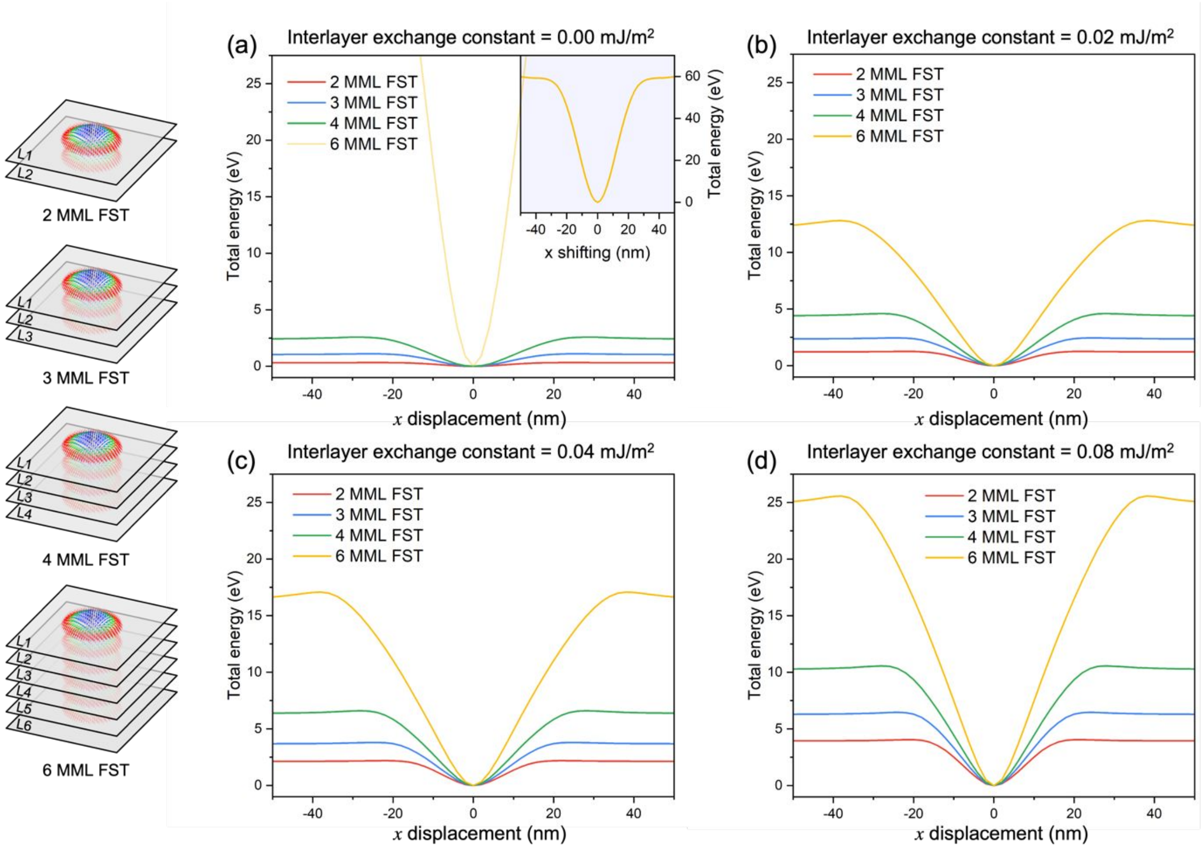

Figure S3. Comparison of the binding energy of 2MML, 3MML, 4MML, and 6MML FSTs with various amplitude of interlayer exchange coupling. The calculated total micromagnetic energy as a function of in-plane displacement of the skyrmion in the top layer of the FSTs with the amplitude of interlayer exchange coupling of (a) 0 mJ m^{-2}, (b) 0.02 mJ m^{-2}, (c) 0.04 mJ m^{-2}, and (d) 0.08 mJ m^{-2}, respectively. The schematic drawing of 2MML to 6MML FSTs are illustrated in the left panel of the figure. The 6 MML FST without interlayer exchange coupling exhibits considerable binding energy, as shown in the inset of (a), which is attributed to the labyrinth domain phase under these parameters.

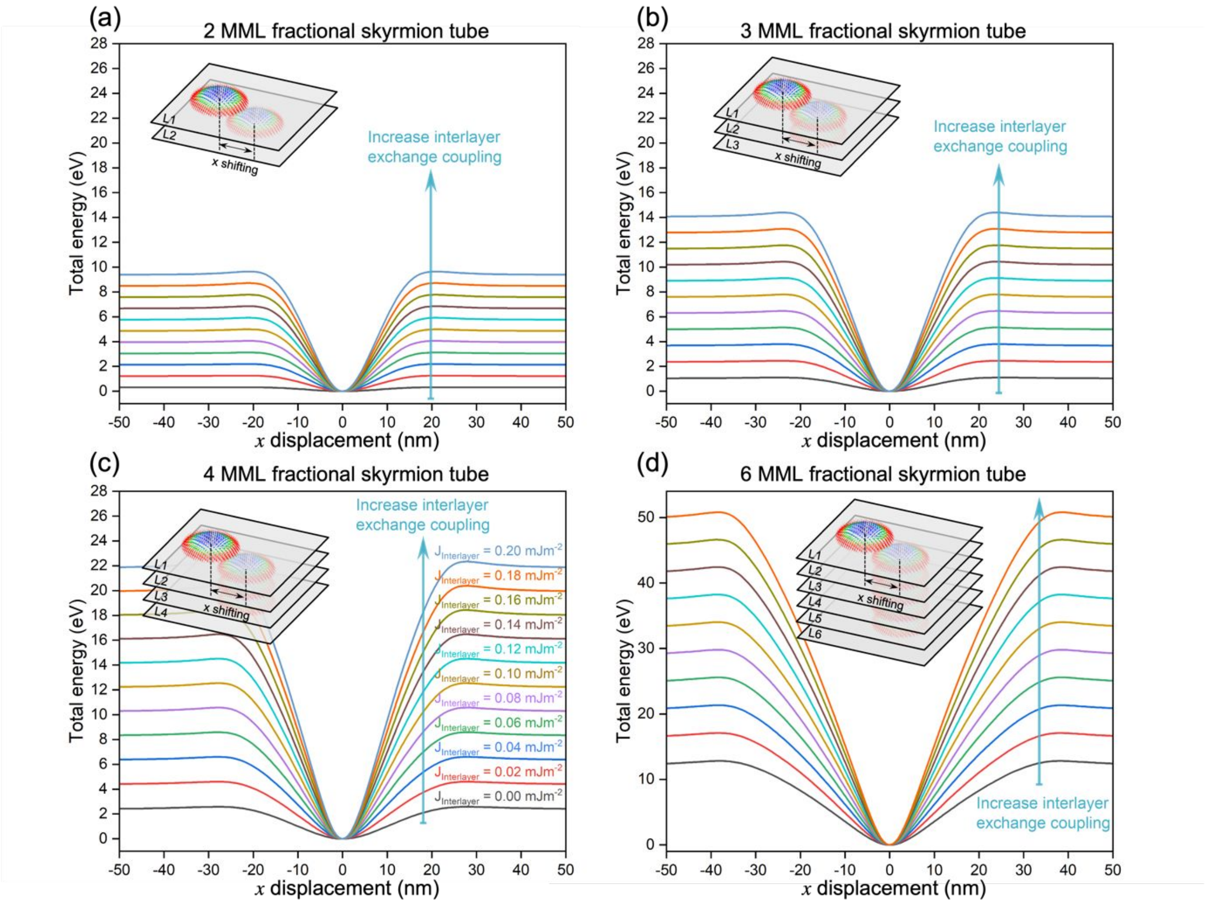

Figure S4. Comparison of the binding energy with various amplitude of interlayer exchange coupling as for (a) 2MML, (b) 3MML, (c) 4MML, and (d) 6MML FSTs, respectively. The solid lines represent the energy change while shifting the top layer skyrmion and the colour code stands for the amplitude of interlayer exchange coupling varying from 0 mJ m^{-2} to 0.2 mJ m^{-2}. Insets (a) to (d) depict the schematic illustration of shifting the top layer skyrmion in FSTs.

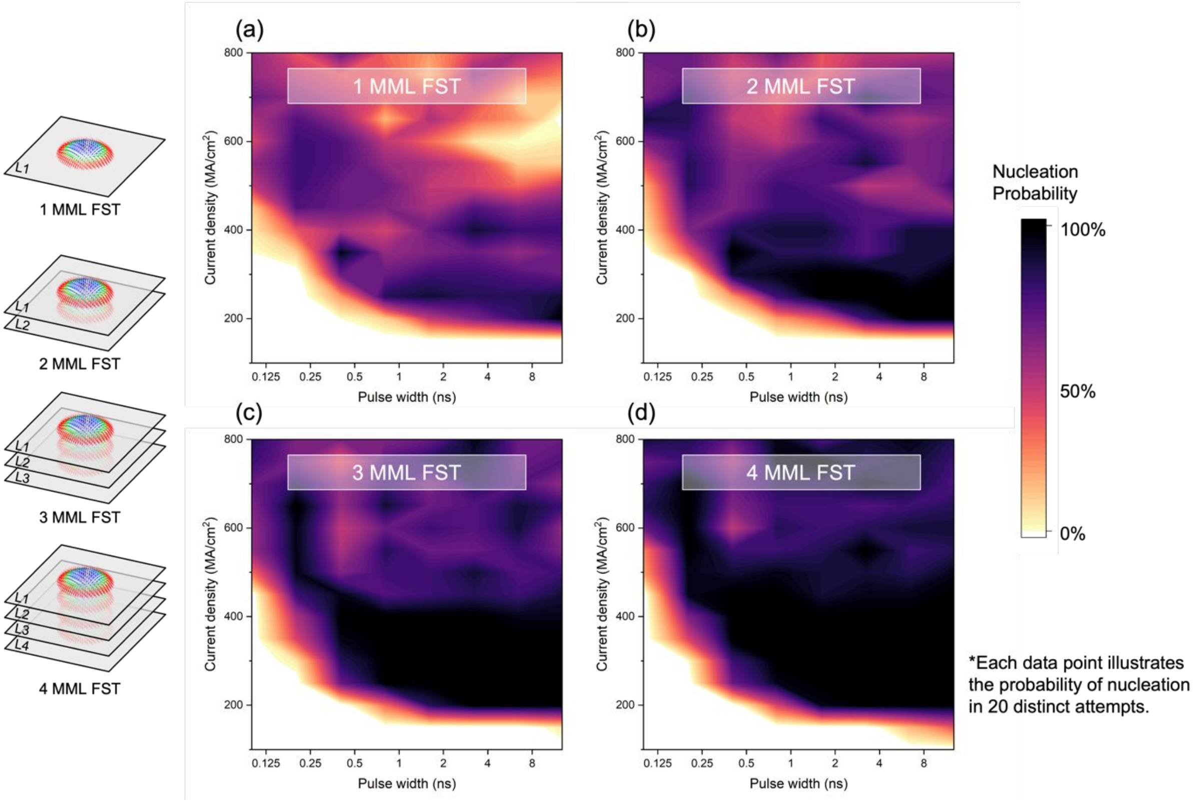

Figure S5. Phase diagram for nucleation probability of (a) 1MML FST, (b) 2MML FST, (c) 3MML FST, and (d) 4MML FST by changing the pulse width of the electric current and the amplitude of the current density under room temperature. The schematic drawing of 1MML to 4MML FSTs are illustrated in the left panel of the figure. The colour code represents the probability of successful nucleation from 0% (white) to 50% (purple) to 100% (black). Each data point illustrates the probability of successful nucleation out of 20 distinct attempts.

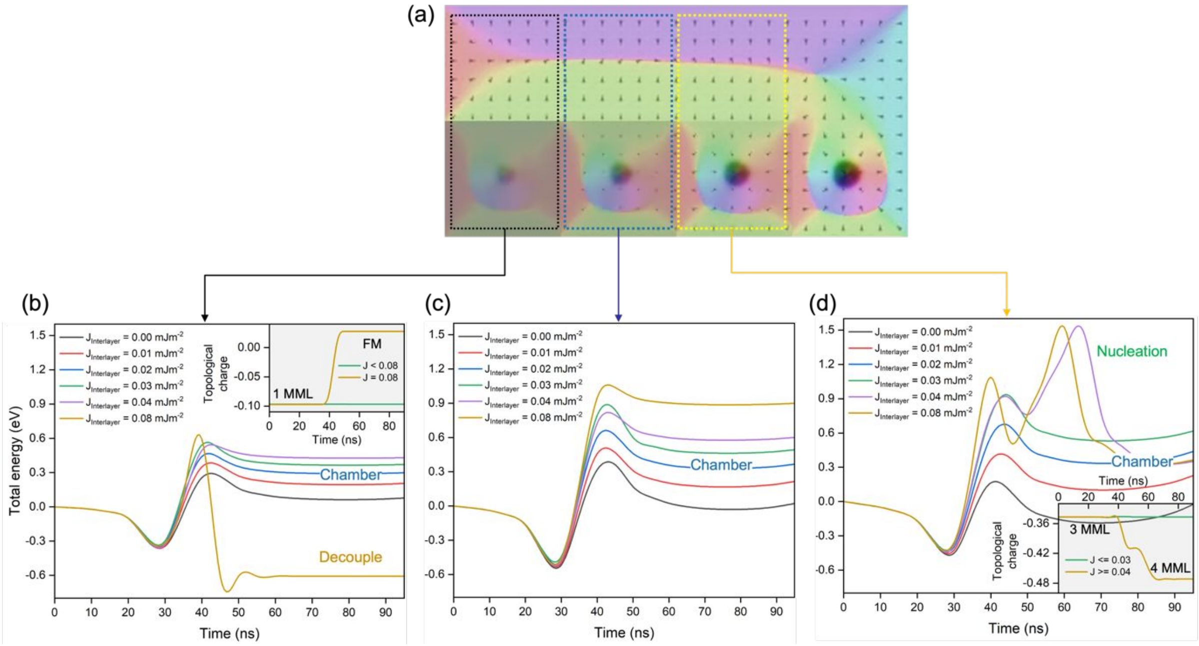

Figure S6. Evolution of the total micromagnetic energy during the chamber process in a 4MML skyrmionic multiplexing device. (a) Illustration of the chamber process in the device. The energy change when (b) a 1MML FST, (c) a 2MML FST, and (d) a 3MML FST is chambered into the 4MML transmission nanotrack. "Decouple" is observed when chambering 1MML FST in (a), and "Nucleation" is observed in chambering a 3MML FST into the main track in (d), while only the "Chamber" phase is observed when chambering the 2MML FST.
